# Relationship between lower-extremity defects and body mass among polish children: a cross-sectional study

**DOI:** 10.1186/s12891-019-2460-0

**Published:** 2019-02-18

**Authors:** Michał Brzeziński, Zbigniew Czubek, Aleksandra Niedzielska, Marek Jankowski, Tomasz Kobus, Zbigniew Ossowski

**Affiliations:** 1 Department of Public Health and Social Medicine, Medical University of Gdansk, Zwycięstwa 42a, 80-210 Gdańsk, Poland; 2 University of Physical Education and Sport in Gdansk, Gdansk, Poland; 3 Gdansk Centre for Health Promotion, Gdansk, Poland; 4 eHealth Solutions Sp. z o.o, Gdansk, Poland

**Keywords:** Children, Body posture, Screening, Flatfoot, Genu valgum, Body mass index

## Abstract

**Background:**

Lower extremity defects in healthy children raises interest of researchers as confirmed by numerous published original and review articles. The relationship between lower extremity postural defects and body mass are unclear as published data are inconclusive. The aim of the present cross-sectional study was to analyse the prevalence of lower extremity defects in a large group of 8- to 12-year-old children; and further to assess the probability of defects associated with values of body mass variables.

**Methods:**

The study included prospective anthropometric measurements data of 6992 children (3476 boys and 3516 girls) from Gdansk (Northern Poland). Standard screening test used in Poland for assessment of lower limb defects were used (intermalleolar or intercondylar distance for knee alignment, linear vertical compass for valgus heel, computer podoscope or classical footprint and measuring the Sztriter-Godunow index for flatfeet). Body mass was assessed with local centile charts and IOTF cutoffs. Prevalence of postural defects was compared with an aid of Pearson’s chi-squared test and Fisher’s exact test. Probability of lower extremities postural defect was estimated on the basis of logistic regression analysis, and expressed as an odds ratio (OR) and its 95.0% CI.

**Results:**

The study demonstrated that cumulative prevalence of lower extremity defects (31.5%) was lower than reported in most published studies, most common defects were valgus heel (21.8%) and valgus knee (14.5%). Boys were significantly more frequently diagnosed with lower limb defects overall (*p* < 0.001), as well as with varus knee, valgus heel, flatfoot of any degree. Limb defects were found in 90,2% of obese children, 25,7% of normal weight and 15,1% of underweight children.

**Conclusions:**

Prevalence of some lower extremities defects seems to be sex specific. Prevalence varied across body weight categories and was rising with the increase of BMI. Increased body mass is correlated with a higher risk of developing lower extremity postural defects in children.

## Background

Posture is defined as the alignment and orientation of the segments of the body when held in the upright position. The biggest importance in quantitative posture evaluation is placed on feet and legs, pelvis, spine, shoulders, and head [1]. Determinants of correct (physiological) stature include normal structure of the locomotor apparatus, in particular, the skeletal and articular system, as well as appropriate functioning of the muscular and nervous systems [2]. All disturbances thereof may contribute to development of postural abnormality and eventually, to established postural defect. While physical activity has a beneficial influence on somatic development at a young age [3], its lack may produce quite an opposite effect. As a result of civilization changes, the prevalence of excessive body mass is increasing in pediatric population [4, 5]. Accordingly the number of musculoskeletal problems in this population is also likely to increase over the next several years and possibly lifespan of its members. Available data show that children with excessive body weight have higher probability of musculoskeletal pain, bone fractures as well as lower quality of life reported on the mobility scale [6, 7] changes within the lower limbs [8, 9] . This effects in acute health issues like pain [7] but also giving a risk of developing health problems in adulthood [10, 11]. The excess body weight influences the foot function and structure by different mechanisms such as biomechanical alterations (as a result of flatfoot), changes in the plantar fat pad, decreased muscle strength as well as changes in gait parameters [12]. Children who are overweight or obese have over 70% likelihood of becoming obese adults [13]. Therefore, the risk of these children developing typical problems of musculoskeletal system in adulthood including degenerative diseases of the musculoskeletal system, osteoarthritis, foot and heel pain, and low back pain syndromes is high [11, 14, 15]. Data about prevalence and association of lower extremities defects and body mass are solid but still not fully conclusive [16, 17]. Available evidence implies that the prevalence of incorrect posture of lower extremities is higher in children with excess body mass [18]. The prevalence of lower extremity defects varies among studies. Pfeiffer et al. [19] showed that 42% of normal weight children, 51% of overweight children, and 62% of obese children had flatfoot based on the assessment of 852 Austrian kindergarten children with three-dimensional laser surface scanner. The difference between normal and overweight children can also be observed in older age groups. A study conducted in Germany [20] showed 20% of 8–11 years -year-old overweight children had flatfoot compared to less than 10% of normal weight children. Similar data were shown in studies performed in Poland [21] and Span [22] among others.

Also studies from non-European countries reported similar results. A study of 7 to 12-year- old children from Northern Taiwan showed that even over 75% of children with obesity can have flatfoot compared to less than 60% in normal weight and less than 50% of underweight children [23]. In all of the presented studies the prevalence of flatfoot was lower in girls than in boys and was decreasing with age – independent of the body mass of the children.

Data about relation between the body mass and knee abnormalities in children are not so scarce. They present higher prevalence of valgus knee in children with increased body mass. Studies from Germany [24], the USA [25] compared this issue on small groups but a large cohort study from Brazil [26] assessed the prevalence of knee valgus to be 27% in obese Brazilian children aged 5–13.

Considering the gaps in existing data we assessed the prevalence of several lower limb defects in a single large population from the perspective and importance of body weight.

Specifically, the aim of this study was to analyse the prevalence of lower extremities defects in a large group of 8- to 12-year-old children, and to assess the relationship associated with excessive or too low body mass. Our study is the largest sample in recent years assessing both the prevalence of and correlation between lower extremities defects and body mass in a population of Caucasian Europeans.

## Methods

### Participants

The paper presents results of 6992 children (3476 boys and 3516 girls) between 8 and 12 years who underwent a screening physical examination between September 2011 and December 2013, within the framework of the Healthy Student screening programme. Table 1. presents the number of the study subjects, stratified be age (year).

**Table 1:** Number of study subjects, stratified by age (year)

Age (years)	Overall	Girls	Boys
	(n)	(n)	(n)
8	571	290	281
9	2120	1104	1016
10	2096	1020	1076
11	1841	927	914
12	373	175	189
Total	6992	3516	3476

The screening was supervised and conducted by the Centre for Promotion of Children’s Health and Fitness in Gdansk (currently: Gdansk Centre for Health Promotion, Gdansk, Poland). The Centre is run and funded by the City of Gdansk, Poland.

The group of 6992 children represents a general population of children in Gdansk, as the screening is performed in over 75% of children in each group yearly. All parents/caregivers gave a written consent for performing the screening. As a result of the procedure all children (parents) were given a comprehensive fact-sheet with information on the health status of the child with recommendations for healthy behaviours and referrals for further evaluation and/or care if needed based on the test results. The study protocol was approved by Independent Bioethics Committee for Scientific Research of Medical University of Gdansk (decision no. NKBBN/228/2012).

The age in years at the time of the evaluation was recorded, rounded to the nearest 0.01 year. Subsequently, age groups were categorized and classified as a whole, as recommended in literature [27].

### Anthropometric data

Anthropometric measurements (body height and body weight) were taken with the children standing upright in the standard anatomical position, barefoot, with the head positioned in the Frankfurt plane. Body height and weight were measured with an electronic scale (Mensor WE150P3, Poland), validated for children. Body height was measured to the nearest 0.001 m and body weight to the nearest 50 g. The scale was calibrated weekly. Data were used to calculate the Body Mass Index (BMI) = body weight (kg) / [body height (m)]^2^. Body weight, body height and the BMI percentiles were assessed using the polish reference system developed at the Children’s Health Memorial Institute, Warszawa, Poland [28].

### Postural defects assessment methods

The knee alignment was measured by measuring the intermalleolar or intercondylar distance, with the use of a ruler graduated in centimeters. Children were measured in the orthostatic position with observation in a posteroanterior direction view, as recommended in literature [29–31]. The distance of 8 cm and over in the distance between the intermalleolar was considered as an indicator of valgus knee, and the distance of 5 cm or more between the intercondylar distance as varus knee in all age groups.

**Fig. 1:**
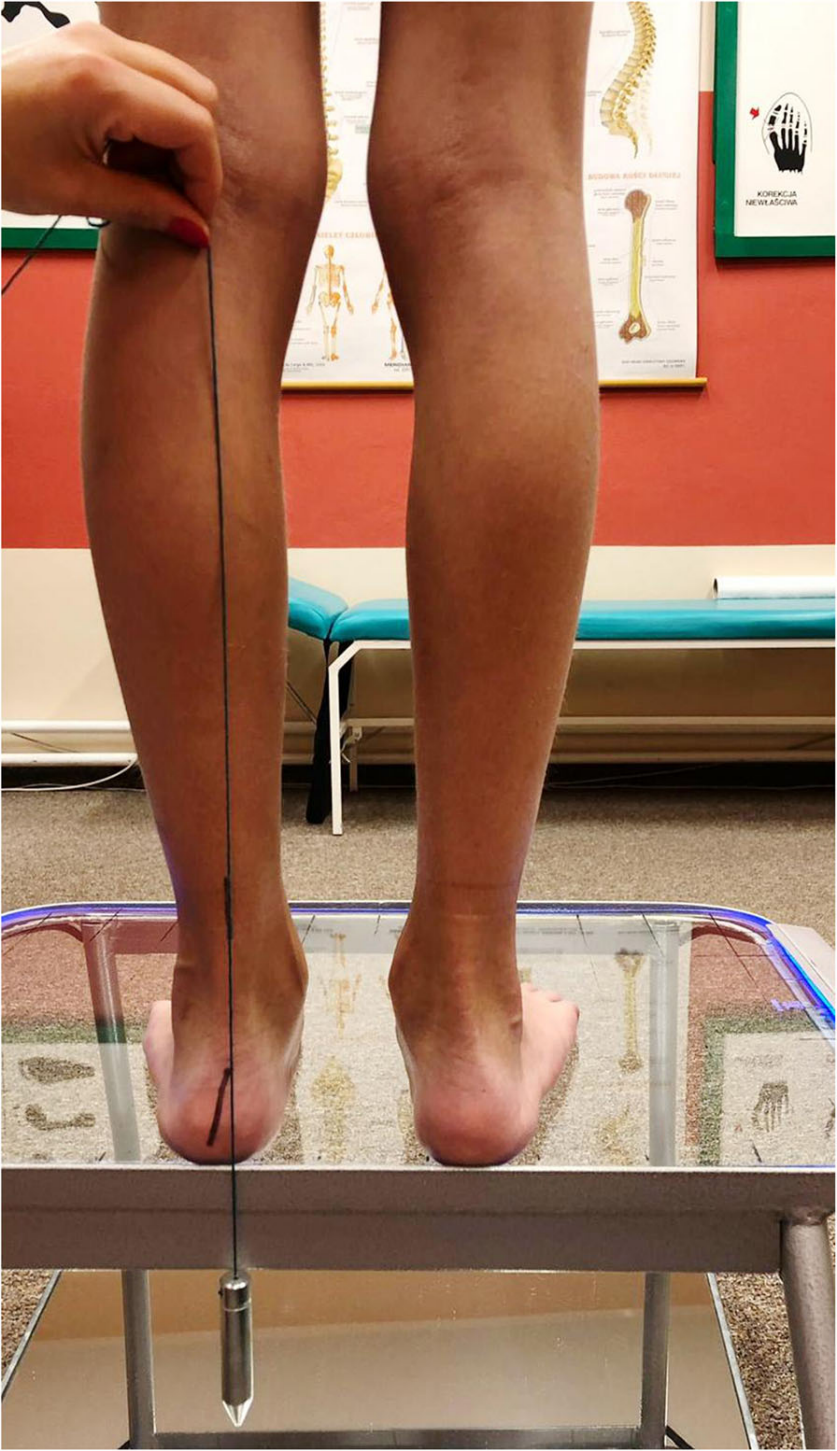
Assessment of valgus heel using linear vertical compass attached to the patients calf

The valgus heel was assessed using a linear vertical compass attached to the patients calf under the knee as presented on Fig. 1. Valgus heel was assessed when the vertical compass was deflecting medially exceeding 1 cm from the knee-heel axis [30].

Flatfoot was assessed by performing a computer podoscope (footprint analysis (device used: Podoscan 2D FootCad, Poland), or a classical footprint and measuring the Sztriter-Godunow index [21]. Flatfoot stage was assessed on the index value. I stage was given for the index value between 0.26–0.49, II to 0.50–0.75, III to 0.76–1.0 and IV index > 1.0 [30].

All measurements were carried out simultaneously by three teams (a physician and a nurse/physiotherapist), who performed all the examinations. Team members were trained and yearly supervised by an internal supervision. For the purpose of the analysis of the association between the prevalence of postural defects and selected somatic parameters (body height, body weight, the BMI), the study subjects were categorized according to their body weight. Obesity, overweight, normal body weight, mild, moderate, and severe underweight were defined in line with the International Obesity Task Force (IOTF) cut-off values [32].

Prevalence of lower extremities postural defects in the study groups was expressed as numbers and percentages, along with their 95% confidence intervals (95% CI). Prevalence of postural defects among girls and boys and weight categories was compared with an aid of Pearson’s chi-squared test and Fisher’s exact test. Probability of lower extremities postural defect associated with a decrease or increase in BMI percentile by one unit was estimated on the basis of logistic regression analysis, and expressed as an odds ratio (OR) and its 95% CI. All calculations were carried out with Statistica 10 package (StatSoft, Tulsa, OK, United States), with the threshold of statistical significance set at *p* < 0.05.

## Results

Out of the examined 6992 children 67.65% were normal weight, 12.84% underweight, 16.3% overweight and 3.2% were obese. There were differences between the prevalence of all types of underweight (higher in girls) and obesity (higher in boys) in the examined group. The results were corresponding to previous studies from this region [33]. Table 2 presents detailed data on the body mass of the participants.

**Table 2:** Body mass distribution in the assessed groups

Body mass (BMI z-score)	Total (%)	Girls (%)	Boys (%)
Severe underweight (z-score ≤ −3)	23 (0,33%)	21 (0,60%)	2 (0,06%) *
Underweight (z-score > −3 ≤ −2)	126 (1,8%)	83 (2,36%)	43 (1,24%) *
Thinness (z-score > −2 ≤ − 1)	749 (10,71%)	430 (12,23%)	319 (9,18%) *
Normal (z-score ≥ −1 < 1)	4730 (67,65%)	2378 (67,63%)	2352 (67,66%)
Overweight (z-score ≥ 1 < 2)	1140 (16,30%)	530 (15,07%)	610 (17,55%)
Obesity (z-score ≥ 2)	224 (3,2%)	74 (2,10%)	150 (4,32%) *

*p < 0,05

In 68.5% of children no lower limbs incorrectness was found. Limb defects were found in nearly every third subject (31.5%); most common defects in this group were valgus heel (21.8%) and valgus knee (14.5%). Prevalence rates for various postural defects in the studied population are presented in Table 3.

**Table 3:** Prevalence of postural defects among 8- to 12-year-old children

Deformities	Whole study group (*n* = 6992)		
	n	%	95% CI
Limb deformities overall^a^	2201	31.5	30.4–32.6
Varus knee	14	0.2	0.1–0.3
Valgus knee	1017	14.5	13.7–15.3
Valgus heel	1526	21.8	20.8–22.8
Flatfoot overall	332	4.7	4.2–5.2
I degree	149	2.1	1.8–2.5
II degree	123	1.8	1.5–2.1
III degree	53	0.8	0.6–1.0
IV degree	7	0.1	0.0–0.2

^a^Children may have more than one limb deformity

Boys significantly more frequently were diagnosed with lower limb defects overall (*p* < 0.001), as well as with varus knee, valgus heel, flatfoot of any degree, I and II degree. Detailed data on the prevalence of postural defects in boys and girls are presented in Table 4.

**Table 4:** Prevalence of postural defects among 8- to 12-year-old children stratified according to sex

Deformities	Girls (*n* = 3516)			Boys (*n* = 3476)			*p*-value
	n	%	95% CI	n	%	95% CI	
Limb deformities overall^a^	1020	29.0	27.5–30.5	**1181**	**34.0**	**32.4–35.6**	**< 0.001**
Varus knee	1	0.0	0.0–0.1	**13**	**0.4**	**0.2–0.7**	**0.001**
Valgus knee	507	14.4	13.2–15.6	510	14.7	13.5–15.9	0.765
Valgus heel	676	19.2	17.9–20.5	**850**	**24.5**	**23.1–26.0**	**< 0.001**
Flatfoot overall	117	3.3	2.7–3.9	**215**	**6.2**	**5.4–7.1**	**< 0.001**
I degree	53	1.5	1.1–2.0	**96**	**2.8**	**2.3–3.4**	**< 0.001**
II degree	40	1.1	0.8–1.5	**83**	**2.4**	**1.9–2.9**	**< 0.001**
III degree	22	0.6	0.4–0.9	31	0.9	0.6–1.3	0.200
IV degree	2	0.1	0.0–0.3	5	0.1	0.0–0.2	0.250

^a^ Children may have more than one limb deformity

Limb defects were most commonly diagnosed in obese children (90.2%) with significantly fewer diagnosed in normal weight (25.7%) and underweight children (15.1%). Detailed data on the prevalence of defects in various weight categories are presented in Table 5.

**Table 5:** Prevalence of specific postural defects among 8- to 12-year-old children, stratified according to the IOTF cut-offs for BMI

Deformities	Normal weight (*n* = 4730)			Severe underweight (*n* = 23)			Moderate underweight (*n* = 126)			Mild underweight (*n* = 749)			Overweight (*n* = 1140)			Obesity (*n* = 224)		
	n	%	95% CI	n	%	95% CI	n	%	95% CI	n	%	95% CI	n	%	95% CI	n	%	95% CI
Limb deformities overall	1217	25.7	24.5–27.0	6	26.1	10.2–48.4	19	15.1	9.3–22.5	113	15.1	12.6–17.8	644	56.5	53.4–59.4	202	90.2	87.6–95.2
Varus knee	8	0.2	0.1–0.4	0	0.0	0.0–14.8	2	1.6	0.2–5.6	3	0.4	0.1–1.2	0	0.0	0.0–0.3	1	0.4	0.0–2.5
Valgus knee	295	6.2	5.5–6.9	2	8.7	1.1–28.0	0	0.0	0.0–2.9	3	0.4	0.1–1.2	524	46.0	43.0–48.9	193	86.2	80.9–90.4
Valgus heel	1021	21.6	20.4–22.8	6	26.1	10.2–48.4	18	14.3	8.7–21.6	107	14.3	11.9–17.0	305	26.8	24.3–29.5	69	30.8	24.8–37.3
Flatfoot overall	185	3.9	3.4–4.5	1	4.3	0.1–21.9	0	0.0	0.0–2.9	9	1.2	0.6–2.3	99	8.7	7.1–10.5	38	17.0	12.3–22.5
I degree	81	1.7	1.3–2.1	0	0.0	0.0–14.8	0	0.0	0.0–2.9	4	0.5	0.1–1.4	49	4.3	3.2–5.6	15	6.7	3.8–10.8
II degree	66	1.4	1.1–1.8	1	4.3	0.1–21.9	0	0.0	0.0–2.9	3	0.4	0.1–1.2	42	3.7	2.7–4.9	11	4.9	2.5–8.6
III degree	32	0.7	0.5–1.0	0	0.0	0.0–14.8	0	0.0	0.0–2.9	2	0.3	0.0–1.0	8	0.7	0.3–1.4	11	4.9	2.5–8.6
IV degree	6	0.1	0.0–0.3	0	0.0	0.0–14.8	0	0.0	0.0–2.9	0	0.0	0.0–0.5	0	0.0	0.0–0.3	1	0.4	0.0–2.5

^1^ Children may have more than one limb deformity. IOTF – International Obesity Task Force

In overweight and obese children valgus knee was the most common postural defect found (46.0 and 86.2% respectively).

Owing to body weight-specific differences in prevalence of various limb defects, we calculated their probability associated with a decrease or increase in the BMI percentile by one unit in relation to the 95th percentile. The increase in the BMI percentile by one unit in relation to the 95th percentile was shown to be associated with a significant (2.0%) increase in the likelihood of any lower limb defects. When analyzed for specific defects, most evident excess risk was found for valgus knee (9.0%), followed by valgus heel (1.0%), flatfoot overall and I-III degree flatfoot (2.0% for each). However, the increase in the BMI percentile was also associated with a significant (3.0%) decrease in the probability of varus knee. Probability of lower limb defects associated with the increase in the BMI percentile by one unit is shown in Table 6.

**Table 6:** Likelihood of postural defect in lower extremities associated with an increase in BMI percentile by one unit in relation to the 95th percentile

Defect	OR	95% CI	*p*
Limb deformities overall	1.02	1.02–1.03	< 0.001
Varus knee	0.97	0.95–0.99	0.009
Valgus knee	1.09	1.08–1.09	< 0.001
Valgus heel	1.01	1.01–1.01	< 0.001
Flatfoot overall	1.02	1.02–1.02	< 0.001
I degree	1.02	1.01–1.03	< 0.001
II degree	1.02	1.02–1.03	< 0.001
III degree	1.02	1.01–1.03	< 0.001
IV degree	1.00	0.98–1.03	0.927

## Discussion

To our knowledge this is one of the largest studies performed in recent years [8, 34] assessing several lower limb defects in children. This study demonstrated that the prevalence of lower limbs postural defects in a population of 8- to 12-year-old children from Gdansk was 31.5% with sex-specific difference.

Prevalence of flatfoot was significantly lower in the current study than reported in most previous studies [8, 9, 23]. The prevalence of flatfoot in obese/overweight children was lower than presented in the study from Taiwan [23], where flatfoot was found in 58.7% of examined children in a similar age group. It is worth noting that not all of the previously published studies could be compared (in the obesity prevalence) due to differences in methodology or the data were from an unrepresentative sample. It is also worth noting that the second study from this region reported lower prevalence of flatfoot in a similar age group (28.0% in a 5–13 year-old group) [9]. However, there are several studies reporting similar results in children aged 5–15, published from different regions of the world regions: Mauch et al. [20] report the percentage of flatfoot at 14.5%, Stavlas et al. [35] reported 5.1% in a group of boys and 3.7% in girls. Similarly, low level of flatfoot (2.7%) was reported in the survey carried out by Garcia-Rodriguez et al. [36]. Our study reports lower results than the previous Polish study by Woźniacka et al. [21]. Flatfoot was observed in 6.2% of girls in both feet and in 12.1% of boys in the left foot and in 11.5% in the right foot – the group differed in age (3-13 year old). Higher prevalence of flatfoot among boys at various stages of somatic development has been reported previously by other authors [6, 15, 19, 34]. However, the reasons behind sex-specific differences in the prevalence of some postural defects remain yet to be established. Mickle et al. suggest that this is due to the difference in the development time of the medial longitudinal arch [37]. Nevertheless, our study is similar to other presenting lower prevalence of flatfoot in girls than in boys and in normal weight than overweight children [9, 21, 23, 35, 36] . This seems to be a well-established relation based on present studies. Newer studies performed in Australia by Evans et al. [34], and by Gijon-Nogueron et all. in Spain [38] using foot posture index (FPI-6) methodology, showed no correlation of body mass and foot posture, but reported similar prevalence of flatfoot overall.

The difference in the prevalence of flatfoot may be associated with different methods used in studies as previously noticed by Evans [34, 39], Chang [23] and others. Moreover, the flatfoot prevalence changes with the aging of children. It is caused by the development of medial longitudinal arch of feet which development should be full by the age of 9 [40]. In our study we only enrolled children above the age of 8. The main focus of our study was not the assessment of age changes but its correlation with body mass.

As Evans stated, there is a need for further analysis in a large groups using a uniform method to assess the reality of anatomically based flatfeet prevalence.

Knee defects were assessed in a smaller number of studies. In those available, the prevalence of valgus and varus knee varies significantly. A Brazilian study by Ciaccia et al. [26] reported the 7.1% prevalence of knee valgus in children aged 7–12. Another Brazilian study found much higher prevalence in of over of valgus knee (56.0%), however, over 60.0% of the examined population was overweight or obese, which could have strongly affected the results [41]. In our study a statistically significant difference in the prevalence of valgus knee in normal, overweight, and obese children was observed, consistent with previous studies [25, 26]. In addition we found sex-specific differences in the prevalence of knee and heel defects, which was in contrast to findings of Walker et al. [42]. Our results show that except for valgus knee all examined limb defects were more frequent in boys. Although the difference is of statistical significance, we need to acknowledge that the crude numbers were very low for both sexes. Differences in fat distribution in boys and girls as well as differences in general limbs development during pubertal age are plausible reasons [24, 42]. Although new study published by Walker et al. using standing radiographs supports the results from clinical measurements and dual-energy X-ray absorptiometry (DEXA) scans [42].

We found no studies reporting the prevalence of valgus heel in this age group based on a population representative sample. We have found a high percentage of children with this defect, which is the most frequently reported defect of all examined defects in this study. It is not recognized in the literature as causing any health complications for children or in their future adolescent and adult life. This assessment is a routine part of the pediatric screening examination of body posture in Poland [30], and probably can be assessed as within normal values based on other methods.

Our study demonstrated that prevalence of defects in lower extremities varied across the IOTF body weight categories, and was the highest among obese children (90.2%). The prevalence of lower extremity defects dropped off with a decrease in the body weight category from 90.2% in obese subjects to 15.1% in those who were underweight. Association between body mass and flatfoot was previously observed in several but not all publications. Chang and all., Pfeifer and all. [19, 23] in their original studies in different age groups confirmed the association. Evans [34, 39] in two studies, did not confirm association, using different method (the Foot Posture Index) in a group of 140 and then 728 Australian children, as well as a study by Gijon-Nogueron in over two thousands children from Spain [38]. Results from systematic reviews are only available for adults and also tend to confirm the connection between obesity and flat foot [15].

In several studies the association between body mass and knee disorders was measured showing a significant difference in the prevalence of knee valgus in obese children [6, 24, 42, 43] but only few were prospective studies based on large groups and comparing obese to normal weight children [24, 44]. To our knowledge this is the largest single centre study assessing that connection.

We present the likelihood of postural defects in lower extremities associated with an increase in the BMI percentile by one unit in relation to the 95th percentile to be used by parents as well as for clinical education purposes. This should be a convenient tool for the assessment of the probability of existing of any limb postural defects in correlation to an increase of the body mass by one centile.

Overweight and obese children without postural defects of lower extremities were a minority in our cohort. This justifies further population-based screening, since its results may constitute an important basis for development of prophylactic programmes in a school setting and for other activities aimed at improving health awareness of the society. Moreover, the results show that limb defects can cause health problems in childhood, adolescence [45, 46] and follow children into adulthood [11], and that prevention/treatment of obesity is one of the most effective solutions [15] to prevent them.

This study has several limitations that need to be taken into consideration:

1. The primary focus of the screening performed at schools was not only postural defect examination but a full assessment of children’s health status. Methods employed were designed to be as simple as possible to be applicable in school environment and settings, therefore, they were screening rather than confirmation methods.
2. Although all of nearly 7000 children were screened by three teams only, they were performing the examinations throughout the period of 2 year. The duration of the study could have influence he medical staff’s ability to assess the posture. On the other hand all team members were well-trained and had been performing screening examinations in schools for several years already and were yearly supervised.
3. Our study employed several methods that are typically used in school screening examinations in Poland. These methods usually involve more manual and traditional assessment than the methods used in studies performed to assess postural defects as a primary outcome measure.
4. Several methods used in our study have been used for 30–40 years when the prevalence of obesity and overweight was much lower both in Poland and worldwide. These methods may not be appropriate for assessment of postural defects in children with obesity, as they may not fully assess the musculoskeletal defects influenced by adipose tissue –which influences the precision of the intermalleolar or intercondylar distance measurement and may present limitations, in the stance only assessment. New methods using more precise equipment or schemes (ex. FPI-6) as well as non-weight-bearing assessment of knee and feet should also be considered in future population studies.

The results of the study and their comparison to the findings described in the present literature can have two-way implications. Firstly, the study should stimulate national recommendations for screening to implement the best known practices/methods to be used in the school screening on a daily basis. On the other hand, it should enforce the real problem of postural defects in the Polish population. It is also worth discussing the validity of screening with traditionally used methods [47]. As several new methods and models of assessment in population screening are presented [21, 34, 39], should we use them instead? More generally, should we perform population screening for foot/posture defects at all? Several authors have expressed a negative opinion and not supported or recommendations towards population screening of body posture defects – limb defects [48] or idiopathic scoliosis [49, 50], which are often performed together. At the center of these discussions and doubts are the health benefits [50, 51] and financial cost of such screenings [52]. These questions have never been assessed in Polish healthcare/school system, where thousands of postural defects examinations are performed in children aged 3–18 yearly. Results of our study are not an answer to those questions, yet showing a high percentage of lower limb defects may signal a need to increase age matched physical activity of both – overweight and normal weight children as a best practices prevention strategy of future musculoskeletal problems.

## Conclusions

The study assesses several lower extremity defects in a large population of children showing that the probability of lower extremity defects increases with the increase in body mass. These data further support prevention and early treatment of overweight in children reducing risks for cardiovascular and metabolic diseases, but also musculoskeletal defects. Nevertheless results of our study should also be viewed relative to the debate on standardizing methods and should we perform postural defect screening at all?

## Abbreviations

BMI: Body Mass Index
DEXA: Dual-energy X-ray absorptiometry
IOTF: International Obesity Task Force
